# Efficacy and Safety of Nivolumab Monotherapy in Patients with High PD-1–Positive CD8/Treg Ratio in Advanced NSCLC and Gastric Cancer: A Phase II, Multicenter Study

**DOI:** 10.1158/2767-9764.CRC-25-0169

**Published:** 2025-10-13

**Authors:** Kohei Shitara, Motohiro Tamiya, Kyoichi Okishio, Hisashi Hosaka, Katsunori Shinozaki, Nobuhiko Seki, Hiroki Hara, Yukiya Narita, Takeshi Shiraishi, Yosuke Tamura, Akihito Tsuji, Kunihiro Tsuji, Naohiro Watanabe, Hiroshi Tanaka, Toshifumi Yamaguchi, Kensei Yamaguchi, Hiroki Izumi, Yasunori Ushida, Hideaki Suna

**Affiliations:** 1 Department of Gastrointestinal Oncology, National Cancer Center Hospital East, Kashiwa, Japan.; 2 Department of Thoracic Oncology, Osaka International Cancer Institute, Osaka, Japan.; 3 Department of Thoracic Oncology, NHO Kinki-Chuo Chest Medical Center, Sakai, Japan.; 4 Department of Gastroenterology, Gunma Prefectural Cancer Center, Gunma, Japan.; 5 Division of Clinical Oncology, Hiroshima Prefectural Hospital, Hiroshima, Japan.; 6 Division of Medical Oncology, Department of Internal Medicine, Teikyo University School of Medicine, Itabashi, Japan.; 7 Department of Gastroenterology, Saitama Cancer Center, Saitama, Japan.; 8 Department of Clinical Oncology, Aichi Cancer Center Hospital, Nagoya, Japan.; 9 Department of Medical Oncology, Japanese Red Cross Matsuyama Hospital, Matsuyama, Japan.; 10 Department of Respiratory Medicine and Thoracic Oncology, Osaka Medical and Pharmaceutical University Hospital, Takatsuki, Japan.; 11 Department of Clinical Oncology, Kagawa University Faculty of Medicine, Miki, Japan.; 12 Department of Medical Oncology, Ishikawa Prefectural Central Hospital, Kanazawa, Japan.; 13 Department of Thoracic Oncology, Aichi Cancer Center Hospital, Nagoya, Japan.; 14 Department of Internal Medicine, Niigata Cancer Center Hospital, Niigata, Japan.; 15 Cancer Chemotherapy Center, Osaka Medical and Pharmaceutical University Hospital, Takatsuki, Japan.; 16 Department of Gastroenterological Chemotherapy, Cancer Institute Hospital of Japanese Foundation for Cancer Research, Koto, Japan.; 17 Department of Thoracic Oncology, National Cancer Center Hospital East, Kashiwa, Japan.; 18 Department of Data Science, Ono Pharmaceutical Co., Ltd., Osaka, Japan.; 19 Oncology Clinical Development Planning Division, Ono Pharmaceutical Co., Ltd., Osaka, Japan.

## Abstract

**Purpose:**

It is challenging to identify the appropriate patients who benefit from anti–PD-1/PD-L1 monotherapy. For predicting effectiveness of anti–PD-1/PD-L1 monotherapy, this open-label phase II study (ONO-4538-88) evaluated the potential of the tumor-infiltrating lymphocyte (TIL) biomarker: the balance between cytotoxic T cells and regulatory T cells.

**Patients and Methods:**

Patients with advanced non–small cell lung cancer (NSCLC) or gastric cancer were screened between March 2021 and January 2022. Eligible patients who met the prespecified TIL biomarker criteria received nivolumab monotherapy. The primary endpoint was objective response rate (ORR). The secondary endpoints included overall survival and progression-free survival. Conventional biomarkers (tumor proportion score, combined positive score, tumor mutation burden, and microsatellite instability) were exploratorily analyzed, and safety was also assessed.

**Results:**

Thirty-seven patients with NSCLC and 127 patients with gastric cancer were eligible for TIL analysis: 6 (16.2%) and 15 patients (11.8%) met the TIL biomarker criteria, respectively; a part of them were assessed. For NSCLC and gastric cancer, the ORR was 80% (4/5 patients) and 36.4% (4/11 patients), respectively; all the five patients and 5/11 patients had a reduction in tumor size, respectively; the median overall survival was not reached and 25 months, respectively; and the median progression-free survival was not reached and 5.59 months, respectively. Treatment-related adverse events occurred in 13/19 patients overall: 5/6 patients for NSCLC and 8/13 patients for gastric cancer.

**Conclusions:**

Although the low positive rate of the TIL biomarker limits interpretation, the promising ORRs suggest signs of the TIL biomarker’s predictability for nivolumab monotherapy.

**Significance:**

In this phase II study, we examined the predictive utility of the TIL biomarker for nivolumab monotherapy. Although the positivity of the TIL biomarker was limited, the promising efficacy and safety profile in the TIL biomarker–positive patients may suggest the potential utility of the TIL biomarker.

## Introduction

Nivolumab, an anti–PD-1 antibody, blocks the interaction between PD-1 on T cells and its ligands (PD-L1 and PD-L2) on tumor cells, enhancing T-cell activity and anticancer effects. It has shown clinical benefits across multiple cancers and is approved in more than 65 countries (1–8). However, the response to PD-1/PD-L1 blockade, including nivolumab, is seen in a limited population, and these therapies can cause long-term, serious immune-related adverse events (AE; ref. 9), emphasizing the need for identifying the right patient groups.

Key biomarkers predicting anti–PD-1/PD-L1 therapy effectiveness include high microsatellite instability (MSI-H), which indicates DNA mismatch repair deficiency (dMMR) and generates numerous neoepitopes, making these tumors more immunogenic and responsive to the treatment. High tumor mutation burden (TMB) also predicts response by increasing tumor-specific antigens that trigger immune reactions. Although both MSI-H/dMMR and high TMB have been validated as biomarkers for anti–PD-1/PD-L1 therapy in advanced cancers, they remain imperfect (10–12). Furthermore, identifying patients responsive to anti–PD-1/PD-L1 therapies despite low MSI, microsatellite stability (MSS), and/or low TMB populations is still necessary.

Whereas tumor PD-L1 expression is also a validated biomarker in some cancer types, its predictive value is mixed (13–15), as PD-1/PD-L1 inhibitors have shown effectiveness in some patients regardless of PD-L1 levels in gastric cancer and non–small cell lung cancer (NSCLC; refs. 2, 5, 16–20). Therefore, there is an unmet need for more reliable biomarkers to guide treatment decisions.

In the tumor microenvironment (TME), PD-1 is expressed on tumor-infiltrating lymphocytes (TIL), including cytotoxic CD8^+^ T cells, the major effector of cancer immunity, and Foxp3^+^ regulatory T (Treg) cells, which counteract the activity of CD8^+^ T cells. Studies have shown that the PD-1–positive rate of CD8^+^ T cells in the TME positively correlates with the effectiveness of PD-1 blockade (21–23), whereas the PD-1–positive rate in Treg cells is associated with resistance against PD-1 blockade (24, 25), suggesting that the balance of PD-1 expression between CD8^+^ T cells and Treg cells in the TME can be a predictive biomarker for the PD-1 blockade therapy. A retrospective cohort study further assessed the hypothesis using flow cytometry assay of TILs from patients with gastric cancer and NSCLC who received PD-1 blockade monotherapy (26) and revealed that the responders for PD-1 blockade therapy indeed had high PD-1 expression in CD8^+^ T cells (≥40%) and a PD-1 expression ratio of CD8^+^ T cells to Treg cells in TILs ≥1. The usefulness of the putative biomarker (hereinafter referred to as the TIL biomarker) needs to be further validated and adapted to wider patient populations in prospective studies. We had focused on both NSCLC and gastric cancer, which have distinct natural histories and responsiveness, because it is possible to apply the results in this study to other cancer types, if we could have shown the usefulness of the TIL biomarker in both NSCLC and gastric cancer.

To evaluate the effectiveness of the TIL biomarker and find the patient population that shows sufficiently high responsiveness to nivolumab monotherapy, we conducted an open-label phase II study (ONO-4538-88) including patients with advanced NSCLC unsuitable for definitive radiation or those with unresectable advanced gastric cancer.

## Materials and Methods

### Study design and patients

This ONO-4538-88 study is a multicenter, open-label, uncontrolled phase II study to evaluate the efficacy and the safety of nivolumab therapy in patients with recurrent or stage IIIB/IIIC/IV NSCLC (according to the eighth edition of the Union for International Cancer Control tumor–node–metastasis classification of malignant tumors) unsuitable for definitive radiation or those with unresectable advanced or recurrent gastric cancer who met all TIL biomarker criteria: (i) PD-1 positivity (%) in CD8^+^ T cells/PD-1 positivity (%) in Treg cells of ≥1 and (ii) PD-1 positivity (%) in CD8^+^ T cells of ≥0. It is crucial to collect data from a broad range of patient populations to estimate a cutoff value for identifying populations with high efficacy of nivolumab monotherapy based on the results of 30 patients each with NSCLC and gastric cancer. Thus, the PD-1 positive rate (%) for CD8^+^ T cells, which was 40% in the retrospective study, was set at 0% at the start of this study. All patients provided written informed consent to participate in this study and agreed to provide tumor tissues for biomarker analysis. The study was approved by institutional review boards and conducted in accordance with the Declaration of Helsinki, the Good Clinical Practice guidelines, and other relevant regulations.

Eligible patients were those ages at least 20 years with pathologically or cytologically diagnosed recurrent NSCLC or stage IIIB/IIIC/IV NSCLC unsuitable for definitive radiation or pathologically diagnosed recurrent or unresectable advanced gastric or esophagogastric junction cancer. Patients had no previous history of systemic anticancer therapy as first-line treatment for progressive or metastatic disease, had at least one measurable lesion defined by the RECIST guideline version 1.1 (27), and had an Eastern Cooperative Oncology Group performance status (ECOG PS) of 0 or 1. Eligible patients were prospectively evaluated for the TIL biomarker [PD-1 positivity (%) in CD8^+^ T cells/PD-1 positivity (%) in Treg cells of ≥1 and PD-1 positivity (%) in CD8^+^ T cells of ≥0], and those who met the TIL biomarker criteria were enrolled.

We excluded patients with NSCLC who had the exon 19 deletion, the L858R mutation in exon 21 of the EGFR, mutations in the BRAF and the MET genes, fusions in the anaplastic lymphoma kinase, the ROS1 and the neurotrophic tropomyosin receptor kinase genes, or nonsquamous NSCLC whose status for EGFR was indeterminate. Patients with gastric cancer with HER2 overexpression were permitted to enroll. In addition, the patients were given the explanation that approved therapy was available if they chose not to participate in this study before providing written informed consent.

Patients received nivolumab therapy (240 mg, intravenously) every 2 weeks until progressive disease (PD) according to the RECIST guideline version 1.1 (27), clinical disease progression, unacceptable toxicities, or investigators’ adjudication. Dose reduction was not allowed. The continuation of nivolumab monotherapy beyond PD was allowed if the tumor progression was not rapid and clinical benefit was still expected (28).

### Biomarker analysis

The previous study (26) identified two formulas for the TIL biomarker as PD-1 positivity (%) in CD8^+^ T cells/PD-1 positivity (%) in Treg cells of ≥ *x* and PD-1 positivity (%) in CD8^+^ T cells of ≥ *y* on the assumption that the true cutoff values (*x* and *y*) depend on cancer types. For this study, the preliminary cutoff values were set at *x* = 1 and *y* = 0 because a certain level of efficacy could be expected based on the result of the previous study. We isolated and analyzed TILs as described previously (29). In brief, tumor biopsy samples from patients were stored in Tumor & Tissue Preservation Reagent (BD biosciences, #664648) at 0°C and digested within 72 hours after biopsy in Tumor & Tissue Dissociation Reagent (BD biosciences, #661563), which is an optimized solution for dissociating TILs. The dissociated cells were then incubated with antibodies (tubes 1 and 2; Supplementary Table S1), including mouse anti-human PD-1 antibody (#564323; RRID: AB_2738745; BD biosciences), and analyzed using flow cytometry system (FACSLyric; BD biosciences). We plotted the PD-1 positivity in the subsets of CD8^+^ T cells and CD4^+^ FoxP3^+^ CD45RA^−^ T (Treg) cells and then calculated whether the sample was positive for the TIL biomarker using the following criteria: PD-1 positivity (%) in CD8^+^ T cells/PD-1 positivity (%) in Treg cells of ≥1 and PD-1 positivity (%) in CD8^+^ T cells of ≥0 for patients with both NSCLC and gastric cancer. When PD-1 positivity (%) in CD8^+^ T cells or Treg cells was 0, 0.01 was to be substituted for 0 in the calculation of PD-1 positivity (%) in CD8^+^ T cells/PD-1 positivity (%) in Treg cells.

For exploratory evaluation using tumor biopsy samples, we performed genetic analysis, such as determining the TMB score and MSI status using FoundationOne CDx (F1CDx; Foundation Medicine Inc.; ref. 30). In addition, we measured the tumor proportion score (TPS) for patients with NSCLC and combined positive score (CPS) for patients with gastric cancer using PD-L1 IHC 28-8 pharmDx (Dako; ref. 31).

### Outcomes

The primary endpoint was objective response rate (ORR) determined by an independent radiologic review committee (IRRC). The secondary endpoints included disease control rate (DCR), best overall response, the change in the sum of the diameters of target tumors, overall survival (OS), and progression-free survival (PFS), each determined by the IRRC. Tumor response was assessed by imaging every 6 weeks according to the RECIST guidelines version 1.1 (27), with the categories of complete response, partial response (PR), and stable disease.

For the safety evaluation, AEs and treatment-related AEs (TRAE) were recorded according to the Common Terminology Criteria for Adverse Events, version 4.0.

### Statistical analysis

Safety was assessed in patients who received at least one dose of nivolumab [the safety set (SAF)]. The full analysis set (FAS) consisted of patients from the SAF for whom efficacy evaluation was possible. The response evaluable set (RES) consisted of patients in the FAS who had a measurable lesion for diagnostic imaging (per the IRRC assessment) at baseline. The 95% confidence intervals (CI) for ORR, DCR, and best overall response were determined by the Clopper–Pearson method, whereas the medians and their corresponding 95% CIs for PFS and OS were determined by the Kaplan–Meier method and the Brookmeyer–Crowley method with log–log transformation. The PFS and OS rates and their 95% CIs at 6, 12, 18, and 24 months after the start of nivolumab therapy were calculated using the Kaplan–Meier method and the Greenwood formula for variance on log–log transformation.

A Bayesian approach was used to calculate the sample size. The simulation results of ORR in a sample size of 30 confirmed that the most likely cutoff value was the same as the true cutoff value that we assumed. Therefore, the sample size was determined to be 30 each for the NSCLC and gastric cancer groups to allow detection of the cutoff values. The outcomes were analyzed individually in the NSCLC and gastric cancer groups.

## Results

### Patient background

**Figure 1.**
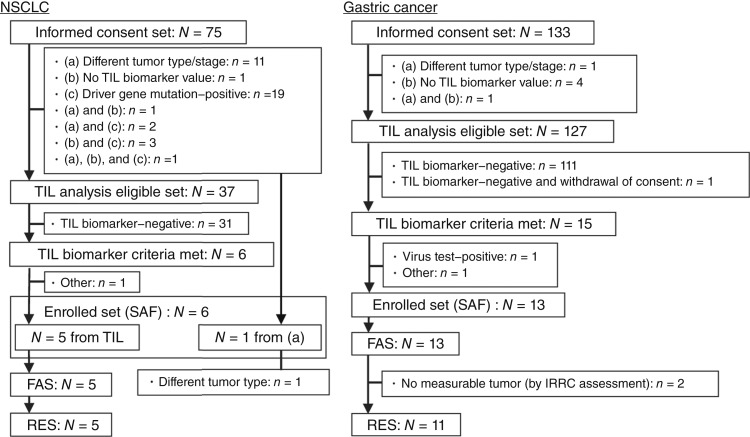
Patient disposition.

**Figure 2.**
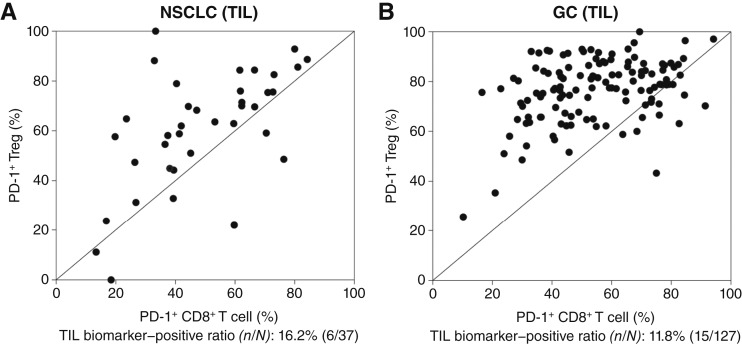
Evaluation of PD-1^+^ Treg cells and PD-1^+^CD8^+^ T cells. **A** and **B,** The scatter plots that show PD-1 positivity (%) in CD8^+^ T cells and Treg cells in patients with (**A**) NSCLC and (**B**) gastric cancer (GC). Straight line indicates PD-1 positivity (%) in CD8^+^ T cells/PD-1 positivity (%) in Treg cells of ≥1.

We screened 75 patients with NSCLC and 133 patients with gastric cancer between March 2021 and January 2022 (Fig. 1). Of those, 37 patients with NSCLC were stage IIIB/IIIC/IV, driver gene mutation–negative, and eligible for obtaining TIL biomarker values and 127 patients had unresectable, advanced, or recurrent gastric cancer and were eligible for obtaining TIL biomarker values (TIL analysis eligible set). The TIL biomarker results were obtained within 4 to 6 days from the biopsy date. Among the TIL analysis eligible set, six patients (16.2%) with NSCLC and 15 patients (11.8%) with gastric cancer had a TIL biomarker value satisfying the eligibility criterion [PD-1 positivity (%) in CD8^+^ T cell/PD-1 positivity (%) in Treg cells ≥1 and PD-1 positivity (%) in CD8^+^ T cells ≥0; Fig. 2]. Six patients with NSCLC and 13 patients with gastric cancer were eventually enrolled and received study treatment at least once, constituting the SAF. One of six patients with a different tumor type was excluded from the TIL analysis eligible set; initially diagnosed with NSCLC, this patient was later identified as having mesothelioma after the start of treatment and was included in the SAF but excluded from the FAS.

In the SAF, the median duration of treatment was 17.99 (range, 0.6–21.7) months in the NSCLC group and 5.13 (range, 0–24.4) months in the gastric cancer group. At the time of the study termination, because of slower accrual than expected (November 2023), all patients with NSCLC and gastric cancer discontinued study treatment as part of this study. The major reason for discontinuation was PD [33.3% (*n* = 2) and 69.2% (*n* = 9) in the NSCLC and gastric cancer groups, respectively]. Two subjects in the NSCLC group and one subject in the gastric cancer group discontinued study treatment but continued nivolumab therapy in the following phase II rollover study (ONO-4538-98; NCT 04566380) as part of an expanded access program.

The FAS included five patients with NSCLC and 13 patients with gastric cancer. Table 1 summarizes the baseline patient characteristics. In the FAS of the NSCLC group (*n* = 5), all patients were men with a median age of 75 (range, 70–78) years. Four patients (80%) had an ECOG PS of 1. The common metastatic site was lymph nodes (*n* = 5; 100%), followed by pleura (*n* = 3; 60%) and pleural effusion (*n* = 3; 60%). In the gastric cancer group (*n* = 13), seven patients were men; the median age was 74 (range, 46–83) years. Five patients (38.5%) had an ECOG PS of 1. The lesion site was the esophagogastric junction in 3 (23.1%) and stomach in 10 (76.9%) patients. The common metastatic sites were the lymph nodes (*n* = 13; 100%), followed by the liver (*n* = 5; 38.5%). The representative of patients with NSCLC and gastric cancer in this study is described in Supplementary Table S2. Supplementary Table S3 shows the list of postprogression anticancer therapies. For NSCLC, two of five patients received the postprogression therapy. One patient received cytotoxic chemotherapy alone, and two patients received targeted therapy plus cytotoxic chemotherapy; of those, one patient received both cytotoxic chemotherapy alone and targeted therapy plus cytotoxic chemotherapy. For gastric cancer, 6 of 13 patients received the postprogression therapy. Four patients received cytotoxic chemotherapy alone, three patients received targeted therapy plus cytotoxic chemotherapy, and one patient received immunotherapy plus cytotoxic chemotherapy; of those, two patients received both cytotoxic chemotherapy alone and targeted therapy plus cytotoxic chemotherapy.

**Table 1.** Patient baseline characteristics (FAS).

Analysis set: FAS	NSCLC *N* = 5 *n* (%)	Analysis set: FAS	Gastric cancer *N* = 13 *n* (%)
Sex	​	Sex	​
Men	5 (100)	Men	7 (53.8)
Women	0	Women	6 (46.2)
Age	​	Age	​
<65 years	0	<65 years	3 (23.1)
≥65 years	5 (100)	≥65 years	10 (76.9)
ECOG PS	​	ECOG PS	​
0	1 (20)	0	8 (61.5)
1	4 (80)	1	5 (38.5)
Organs with metastases	​	HER2	​
Bone	2 (40)	Positive	1 (7.7)
Brain	1 (20)	Negative	12 (92.3)
Lymph node	5 (100)	Lesion sites	​
Pleura	3 (60)	Esophagogastric junction	3 (23.1)
Pleural effusion	3 (60)	Stomach	10 (76.9)
Crus of diaphragm	1 (20)	Gross type (Borrmann classification)	​
Histologic classification	​	Advanced carcinoma type I	2 (15.4)
Adenocarcinoma	3 (60)	Advanced carcinoma type II	0
Squamous cell carcinoma	1 (20)	Advanced carcinoma type III	9 (69.2)
Other	1 (20)	Advanced carcinoma type IV	2 (15.4)
PD-L1 (TPS)	​	Histologic types (Lauren criteria)	​
<1%	1 (20)	Intestinal type	6 (46.2)
≥1%	4 (80)	Diffuse type	5 (38.5)
<50%	1 (20)	Unknown	2 (15.4)
≥50%	4 (80)	Organs with metastases	​
TMB	​	Liver	5 (38.5)
<10 mut/Mb	3 (60)	Lung	1 (7.7)
≥10 mut/Mb	0	Lymph node	13 (100)
Missing	2 (40)	Peritoneum	2 (15.4)
​	​	Pleura	1 (7.7)
		Retroperitoneum	1 (7.7)
		Pleural effusion	2 (15.4)
		PD-L1 (CPS)	​
		<1	1 (7.7)
		≥1	12 (92.3)
		<10	3 (23.1)
		≥10	10 (76.9)
		MSI status	​
		MSS (stable)	7 (53.8)
		MSI-L (low)	0
		MSI-H (high)	2 (15.4)
		Missing	4 (30.8)
		TMB	​
		<10 mut/Mb	5 (38.5)
		≥10 mut/Mb	4 (30.8)
		Missing	4 (30.8)

### Efficacy

The tumor response was assessed in the RES, which included five patients with NSCLC and 11 patients with gastric cancer. The ORR according to the IRRC assessment was 80% in the NSCLC group (95% CI, 28.4%–99.5%; 4 PRs) and 36.4% in the gastric cancer group (95% CI, 10.9%–69.2%; 1 complete response and 3 PRs; Table 2). The DCR as per the IRRC assessment was 80% in the NSCLC group (95% CI, 28.4%–99.5%) and 63.6% in the gastric cancer group (95% CI, 30.8%–89.1%).

**Table 2.** Summary of responses (RES).

Analysis set: RES	NSCLC *N* = 5 *n* (%)	Gastric cancer *N* = 11 *n* (%)
ORR (CR + PR)	4 (80)	4 (36.4)
(95% CI)^a^	(28.4, 99.5)	(10.9, 69.2)
DCR (CR + PR + SD)	4 (80)	7 (63.6)
(95% CI)^a^	(28.4, 99.5)	(30.8, 89.1)
BOR	​	​
CR	0	1 (9.1)
PR	4 (80)	3 (27.3)
SD	0	3 (27.3)
PD	1 (20)	4 (36.4)
NE	0	0

Abbreviations: BOR, best overall response; CR, complete response; NE, not evaluable; SD, stable disease.

a Estimated using the Clopper–Pearson method.

**Figure 3.**
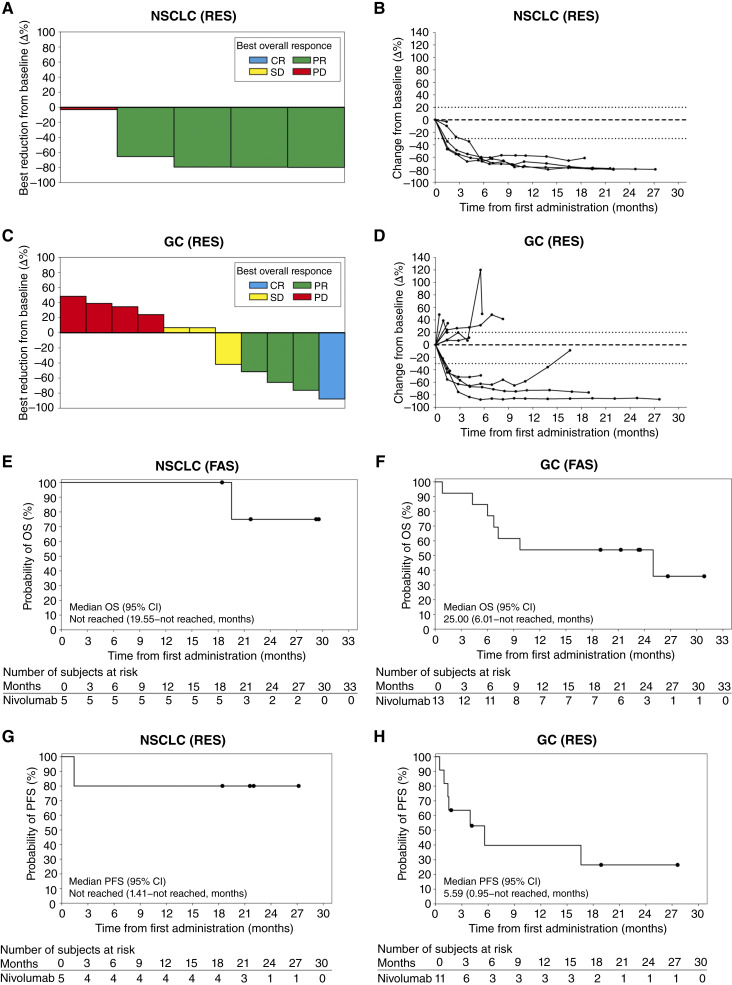
Tumor response, OS, and PFS. **A** and **B,** A summary of (**A**) best reduction and (**B**) percent change from baseline in the sum of the diameters of target tumors in each patient with NSCLC treated with nivolumab. **C** and **D,** A summary of (**C**) best reduction and (**D**) percent change from baseline in the sum of the diameters of target tumors in each patient with gastric cancer (GC) treated with nivolumab. **E** and **F,** A summary of the probability of OS in patients with (**E**) NSCLC and (**F**) GC. **G** and **H,** A summary of the probability of PFS in patients with (**G**) NSCLC and (**H**) GC. The 95% CI was determined by the Brookmeyer–Crowley method with log–log transformation. Circles indicate censoring. CR, complete response; SD, stable disease.

In the NSCLC group (RES), all five patients had a reduction in the tumor size, with four patients having a >50% reduction, which was sustained for >12 months (assessed by the IRRC; Fig. 3A and B). In the gastric cancer group (RES), five patients had a reduction in tumor size (assessed by the IRRC; Fig. 3C), including four patients with a >50% reduction, three of whom had a sustained reduction for >12 months (Fig. 3D).

Regarding OS (FAS) and PFS (RES), in the NSCLC group, the median OS was not reached (95% CI, 19.55 months–not reached; Fig. 3E), and the OS rate was 100% (95% CI, 100%–100%) at 6 to 18 months and 75% (95% CI, 12.8%–96.1%) at 24 months. In the gastric cancer group, the median OS was 25 months (95% CI, 6.01 months–not reached; Fig. 3F), and the OS rate was 84.6% (95% CI, 51.2%–95.9%) at 6 months and 53.8% (95% CI, 24.8%–76%) at 12 to 24 months. In the NSCLC group, the median PFS as per the IRRC assessment was not reached (95% CI, 1.41 months–not reached; Fig. 3G), and the NSCLC PFS rate was 80% (95% CI, 20.4%–96.9%) at 6 to 24 months. In the gastric cancer group, the median PFS by IRRC assessment was 5.59 months (95% CI, 0.95 months–not reached; Fig. 3H), and the PFS rate was 39.8% (95% CI, 11%–68%) at 6 and 12 months and 26.5% (95% CI, 4.4%–56.9%) at 18 and 24 months.

### The relationship between the TIL biomarker and other biomarkers

As *ad hoc* exploratory analyses, we measured conventional biomarkers used for estimating the efficacy of PD-1 and PD-L1 blockade therapy, such as TPS, CPS, TMB, and MSI (Table 3).

**Table 3.** Summary of conventional biomarkers (RES, *ad hoc* analysis).

Analysis set: RES	Category	NSCLC *N* = 5	Analysis set: RES	Category	Gastric cancer *N* = 11
Biomarker		ORR (*n*/*N*)	Biomarker		ORR (*n*/*N*)
PD-L1 (TPS)	TPS <1%	0% (0/1)	PD-L1 (CPS)	CPS <1	0% (0/1)
	TPS ≥1%	100% (4/4)		CPS ≥1	40% (4/10)
	1%≤ TPS <50%	—		1≤ CPS <10	—
	TPS <50%	0% (0/1)		CPS <10	0% (0/1)
	TPS ≥50%	100% (4/4)		CPS ≥10	40% (4/10)
TMB	TMB <10 mut/Mb	66.7% (2/3)	TMB	TMB <10 mut/Mb	33.3% (1/3)
	TMB ≥10 mut/Mb	—		TMB ≥10 mut/Mb	50% (2/4)
	Missing	100% (2/2)		Missing	25% (1/4)
​	​	​	MSI status	MSS	40% (2/5)
				MSI-H	50% (1/2)
				Missing	25% (1/4)

In patients with NSCLC (RES; *n* = 5), one, zero, and four patients were TPS of <1%, TPS of 1%≤ to <50%, and TPS of ≥50%, respectively. The best response of one patient with TPS of <1% was PD, and all four patients with TPS of ≥50% were responders (100%). In the TMB analysis, we also found that the RES of the NSCLC group included three patients with TMB of <10 mut/Mb and two with missing data. The ORR in patients with TMB of <10 mut/Mb was 66.7% (2/3 patients).

In the patients with gastric cancer (RES; *n* = 11), 1, 0, and 10 patients were CPS of <1, CPS of 1≤ to <10, and CPS of ≥10, respectively. The best response of one patient with CPS of <1 was PD, and 4 of 10 patients with CPS of ≥10 were responders (40%). The gastric cancer group included three patients with TMB of <10 mut/Mb and four with TMB of ≥10 mut/Mb, but four had missing data due to poor quality of sample conditions or technical problems. The ORR was 33.3% for a TMB of <10 mut/Mb (1/3 patients) and 50% for a TMB of ≥10 mut/Mb (2/4 patients). The gastric cancer group also included five patients with MSS and two with MSI-H, and four had missing data as described above. The ORR was 40% for MSS (2/5 patients) and 50% for MSI-H (1/2 patients).

### Safety

As shown in Table 4, all 19 patients (SAF; *n* = 6 in the NSCLC and *n* = 13 in the gastric cancer) experienced AEs. TRAEs occurred in 13 patients (68.4%): grade 3 to 4 TRAEs occurred in three patients [pemphigoid and chronic kidney disease (one patient), blood creatine phosphokinase increased (one patient), and liver disorder (one patient)]. There was no fatal grade 5 TRAE. A serious TRAE of pemphigoid occurred in one patient. TRAEs leading to interruption of nivolumab therapy were encountered in three patients [15.8%; blood creatinine increased, chronic kidney disease, and pemphigoid (one patient), diarrhea (one patient), and blood creatine phosphokinase increased (one patient)]. A TRAE leading to discontinuation of nivolumab therapy was identified in one patient (liver disorder). The most frequent TRAEs were rash and diarrhea (three patients; 15.8% each). The incidence of AEs and TRAEs and details of TRAEs in patients with NSCLC and gastric cancer are also tabulated in Table 4.

**Table 4.** Summary of AEs and TRAEs (SAF).

Analysis set: SAF	All		NSCLC		Gastric cancer	
	*N* = 19		*N* = 6		*N* = 13	
	Any grade	Grades 3–5	Any grade	Grades 3–5	Any grade	Grades 3–5
	*n* (%)	*n* (%)	*n* (%)	*n* (%)	*n* (%)	*n* (%)
AEs	19 (100)	11 (57.9)	6 (100)	3 (50)	13 (100)	8 (61.5)
Serious AEs	6 (31.6)	5 (26.3)	3 (50)	2 (33.3)	3 (23.1)	3 (23.1)
AEs leading to interruption of nivolumab therapy	5 (26.3)	4 (21.1)	3 (50)	2 (33.3)	2 (15.4)	2 (15.4)
AEs leading to discontinuation of nivolumab therapy	3 (15.8)	3 (15.8)	0	0	3 (23.1)	3 (23.1)
AEs leading to death^a^	1 (5.3)	1 (5.3)	0	0	1 (7.7)	1 (7.7)
TRAEs	13 (68.4)	3 (15.8)	5 (83.3)	1 (16.7)	8 (61.5)	2 (15.4)
Serious TRAEs	1 (5.3)	1 (5.3)	1 (16.7)	1 (16.7)	0	0
TRAEs leading to interruption of nivolumab therapy	3 (15.8)	2 (10.5)	2 (33.3)	1 (16.7)	1 (7.7)	1 (7.7)
TRAEs leading to discontinuation of nivolumab therapy	1 (5.3)	1 (5.3)	0	0	1 (7.7)	1 (7.7)
TRAEs leading to death	0	0	0	0	0	0
Total TRAEs	13 (68.4)	3 (15.8)	5 (83.3)	1 (16.7)	8 (61.5)	2 (15.4)
Skin and subcutaneous tissue disorders	8 (42.1)	1 (5.3)	4 (66.7)	1 (16.7)	4 (30.8)	0
Rash	3 (15.8)	0	2 (33.3)	0	1 (7.7)	0
Dermatitis acneiform	2 (10.5)	0	0	0	2 (15.4)	0
Rash maculopapular	2 (10.5)	0	1 (16.7)	0	1 (7.7)	0
Blister	1 (5.3)	0	1 (16.7)	0	0	0
Dry skin	1 (5.3)	0	1 (16.7)	0	0	0
Erythema multiforme	1 (5.3)	0	0	0	1 (7.7)	0
Ingrowing nail	1 (5.3)	0	1 (16.7)	0	0	0
Pemphigoid^b^	1 (5.3)	1 (5.3)	1 (16.7)	1 (16.7)	0	0
Pruritus	1 (5.3)	0	1 (16.7)	0	0	0
Investigations	4 (21.1)	1 (5.3)	2 (33.3)	0	2 (15.4)	1 (7.7)
Alanine aminotransferase increased	1 (5.3)	0	1 (16.7)	0	0	0
Aspartate aminotransferase increased	1 (5.3)	0	1 (16.7)	0	0	0
Blood creatine phosphokinase increased^c^	1 (5.3)	1 (5.3)	0	0	1 (7.7)	1 (7.7)
Blood creatinine increased^c^	1 (5.3)	0	1 (16.7)	0	0	0
Neutrophil count decreased	1 (5.3)	0	0	0	1 (7.7)	0
Gastrointestinal disorders	3 (15.8)	0	2 (33.3)	0	1 (7.7)	0
Diarrhea^d^	3 (15.8)	0	2 (33.3)	0	1 (7.7)	0
Constipation	1 (5.3)	0	1 (16.7)	0	0	0
General disorders and administration site conditions	3 (15.8)	0	1 (16.7)	0	2 (15.4)	0
Malaise	2 (10.5)	0	1 (16.7)	0	1 (7.7)	0
Edema	1 (5.3)	0	0	0	1 (7.7)	0
Musculoskeletal and connective tissue disorders	3 (15.8)	0	1 (16.7)	0	2 (15.4)	0
Myalgia	2 (10.5)	0	1 (16.7)	0	1 (7.7)	0
Arthralgia	1 (5.3)	0	0	0	1 (7.7)	0
Endocrine disorders	2 (10.5)	0	0	0	2 (15.4)	0
Hypopituitarism	1 (5.3)	0	0	0	1 (7.7)	0
Hypothyroidism	1 (5.3)	0	0	0	1 (7.7)	0
Respiratory, thoracic, and mediastinal disorders	2 (10.5)	0	1 (16.7)	0	1 (7.7)	0
Epistaxis	1 (5.3)	0	0	0	1 (7.7)	0
Pneumonitis	1 (5.3)	0	1 (16.7)	0	0	0
Hepatobiliary disorders	1 (5.3)	1 (5.3)	0	0	1 (7.7)	1 (7.7)
Liver disorder^e^	1 (5.3)	1 (5.3)	0	0	1 (7.7)	1 (7.7)
Infections and infestations	1 (5.3)	0	0	0	1 (7.7)	0
Gastroenteritis	1 (5.3)	0	0	0	1 (7.7)	0
Nervous system disorders	1 (5.3)	0	0	0	1 (7.7)	0
Peripheral sensory neuropathy	1 (5.3)	0	0	0	1 (7.7)	0
Renal and urinary disorders	1 (5.3)	1 (5.3)	1 (16.7)	1 (16.7)	0	0
Chronic kidney disease^c^	1 (5.3)	1 (5.3)	1 (16.7)	1 (16.7)	0	0

a AE: hematemesis.

b Serious TRAE/TRAE leading to interruption of nivolumab therapy.

c TRAE leading to interruption of nivolumab therapy.

d TRAE that one of two patients with NSCLC had interruption of nivolumab therapy.

e TRAE leading to discontinuation of nivolumab therapy.

## Discussion

This ONO-4538-88 study evaluated the usefulness of the TIL biomarker, defined as PD-1 positivity (%) in CD8^+^ T cell/PD-1 positivity (%) in Treg cells ≥*x* and PD-1 positivity (%) in CD8^+^ T cells ≥*y*, for nivolumab monotherapy in recurrent or advanced NSCLC and gastric cancer. Although nivolumab and chemotherapy have been approved for both NSCLC and gastric cancer recently, we aimed to establish a less toxic therapeutic strategy with nivolumab monotherapy. We first screened patients who met the TIL biomarker value with cutoff values of *x* = 1 and *y* = 0 and identified 6 patients with NSCLC and 15 patients with gastric cancer with values above the cutoff; 5 patients with NSCLC and 13 patients with gastric cancer were enrolled for evaluating efficacy (FAS). As we could not enroll a sufficient number of patients because of slower accrual than planned, we were not able to conclusively assess the predictive utility of the TIL biomarker nor to determine clinically meaningful cutoff values for each cancer type, therefore limiting the overall interpretation.

Although the sample size was small, the IRRC-assessed ORRs was 80% for the NSCLC group (4/5) and 36.4% for the gastric cancer group (4/11) in the enrolled and IRRC-assessed patients who met TIL biomarker criteria. The ORRs for this study exceeded the ORRs for the previous studies with PD-1/PD-L1 blockade monotherapy: about 20% to 30% for advanced, metastatic, or recurrent NSCLC (all-comer population or TPS of ≥1%; refs. 17, 32–34) and about 10% to 15% for advanced gastric cancer (all-comer or CPS of ≥1; refs. 5, 35, 36). In addition, the median OS and PFS were not reached for the NSCLC group and were 25 and 5.59 months, respectively, in the gastric cancer group, which also exceeded the median OS and PFS in the previous studies: about 10 to 20 and 2 to 6 months for NSCLC (17, 32–34, 37) and about 5 to 15 and 1.5 to 2 months for gastric cancer, respectively (5, 35, 36). The results suggest that the TIL biomarker may be associated with the efficacy of nivolumab monotherapy, as indicated in the previous retrospective study (26).

In addition, there was a substantial difference between the median OS of 25 months and PFS of 5.59 months in the gastric cancer group. A previous prospective observational study showed that in patients with gastric cancer treated with chemotherapy after nivolumab monotherapy as a late-line treatment, the median OS was 7.5 months and the median PFS was 2.9 months (38), suggesting a synergistic antitumor effect. Indeed, in this study, 6 of 13 patients with gastric cancer received postprogression therapies containing cytotoxic chemotherapy. Thus, there is a possibility that the TIL biomarker may be prognostic, but the difference might be affected by the subsequent chemotherapy.

Biomarker analysis indicated that the IRRC-assessed patients with NSCLC (*n* = 5) and gastric cancer (*n* = 11) who met the TIL biomarker criteria included four patients with TPS of ≥50% and 10 patients with CPS of ≥10, respectively, and all the patients who responded to nivolumab therapy had a TPS of ≥50% in NSCLC (4/4 patients) and CPS of ≥10 in gastric cancer (4/10 patients). Previous studies have shown that the ORR in the patients with PD-L1–high tumor was higher than in the general population. The ORR for the first-line pembrolizumab monotherapy was 44.8% in patients with NSCLC with TPS ≥50% and 25% in patients with gastric cancer with CPS ≥10 (36, 39). Compared with these previous studies, the ORR of patients with TIL biomarker positivity was numerically even higher (80% for NSCLC and 36.4% for gastric cancer) in this study. Although the sample size is small, it is possible that the TIL biomarker can identify patient populations with higher responsiveness to nivolumab monotherapy among those with high tumor PD-L1 expression.

Regarding patients with conventional biomarker negativity, the previous trials demonstrated that the ORR was 18.8% in patients with NSCLC with TMB <175 mut/exome and 6.7% in those with gastric cancer with TMB <10 mut/Mb (pembrolizumab monotherapy; refs. 36, 40). In this study, three patients each in the NSCLC (*n* = 5) group and the gastric cancer (*n* = 11) group had a TMB of <10 mut/Mb. The ORRs for TMB <10 mut/Mb were 66.7% (2/3 patients) and 33.3% (1/3 patients) in patients with NSCLC and gastric cancer, respectively, which were numerically higher compared with the previous studies. In addition, the ORR was 40% (2/5) in patients with gastric cancer with MSS, which was also numerically higher than that in previous study in patients with gastric cancer with non–MSI-H and CPS >1 (12.4%, pembrolizumab monotherapy; ref. 36). Although we should carefully note that the groups of patients with TIL biomarker positivity had small sample size and contains cases with high tumor PD-L1 expression, the TIL biomarker may help in detecting wider patient populations by testing patients with conventional biomarker-negative such as TMB <10 mut/Mb and MSS. Further investigation with larger sample size would be needed.

The incidence of any TRAEs (83.3% for NSCLC and 61.5% for gastric cancer) seemed numerically higher than those observed in other studies with all-comer populations (58%–71% for NSCLC and 43–60.2 for gastric cancer; refs. 1, 2, 5, 35–37, 41), whereas the incidences of grade 3 to 4 TRAEs (16.7% for NSCLC and 15.4% for gastric cancer) were comparable with those in other studies (7%–18% for NSCLC and 10%–17.8% for gastric cancer; refs. 1, 2, 5, 35–37, 41), and no new safety concerns were identified. By contrast, in certain patient subgroups that were expected to have higher efficacy of anti–PD-1/PD-L1 monotherapy, the incidence of TRAEs tended to be higher; the incidence of TRAEs was 73.4% in patients with NSCLC with PD-L1 ≥50% (pembrolizumab monotherapy; ref. 39) and 70% in patients with colorectal cancer with MSI-H and dMMR (nivolumab monotherapy; ref. 42), which were comparable with that observed in this study. Because the sample size was too small, we hardly concluded the relationship between the higher incidence of TRAEs and higher efficacy in this study.

The percentage of subjects who met the TIL biomarker criteria in this study (positive rates of the TIL biomarker; 16.2% for NSCLC and 11.8% for gastric cancer) was markedly lower than that in the previous retrospective study (60% for NSCLC and 29.2% for gastric cancer, from discovery cohort data), although the cutoff value for enrollment that we set in this study [PD-1 positivity (%) in CD8^+^ T cells ≥0] was broader than that of the responder group in the previous study [PD-1 positivity (%) in CD8^+^ T cells ≥40; ref. 26]. There are some methodologic differences. With respect to the study design, the previous study was a retrospective study, whereas this study is prospective. In addition, although the method for isolating TILs was generally similar (29), the temperature at which tumor samples were preserved was different; 0°C in this study and on ice in the previous study. Those differences may have also led to the lower TIL biomarker positivity rate. Although it is possible that the cutoff values need to be calibrated for each target patient population, the sampling method itself was feasible for multicenter studies and actual clinical practice, and even a biomarker with a low positive rate will be of benefit if it accurately predicts treatment outcomes. Thus, the findings of this study suggest that further assessment of the performance of the TIL biomarker is warranted.

Our study has several limitations. First, the number of enrolled patients who met the criteria for the TIL biomarker was small in both NSCLC and gastric cancer groups, which limited the interpretation of efficacy results and prevented calibration of clinically meaningful cutoff values. Missing data for TMB and MSI in several patients also limited the interpretation of results from the exploratory analyses. In addition, we did not measure other important biomarkers such as Epstein–Barr virus status, which is also known to be related to PD-L1 expression in gastric cancer (43, 44).

In conclusion, although the low positive rate of the TIL biomarker limits interpretation, the ORR, OS, and PFS in the TIL biomarker–positive patients were numerically greater than the previously reported values in all-comer populations. Additionally, the TIL biomarker–positive patients who responded to nivolumab monotherapy included patients who were conventional biomarker–negative, including MSS and TMB <10 mut/Mb. Although the overall results suggest the signs of the TIL biomarker’s predictability for patients who will respond to nivolumab monotherapy, further studies are required to validate the performance of this biomarker and to adapt it to clinical practice.

## Supplementary Material

Supplementary Table S1 The antibodies list used for TIL analysis

Supplementary Table S2 Representativeness of study participants

Supplementary Table S3 Post-progression anti-cancer therapy of the nivolumab monotherapy

## Data Availability

Any researcher may request Ono Pharmaceutical Co., Ltd. to disclose individual patient‐level data from clinical studies through Vivli (https://vivli.org/). Interested researchers should consult https://www.ono-pharma.com/en/company/policies/clinical_trial_data_transparency_policy.html for more information on requests for patient-level data from Ono Pharmaceutical Co., Ltd.

## References

[1] Borghaei H , Paz-Ares L, Horn L, Spigel DR, Steins M, Ready NE, . Nivolumab versus docetaxel in advanced nonsquamous non–small-cell lung cancer. N Engl J Med 2015;373:1627–39. 26412456 10.1056/NEJMoa1507643 PMC5705936

[2] Brahmer J , Reckamp KL, Baas P, Crinò L, Eberhardt WEE, Poddubskaya E, . Nivolumab versus docetaxel in advanced squamous-cell non–small-cell lung cancer. N Engl J Med 2015;373:123–35. 26028407 10.1056/NEJMoa1504627 PMC4681400

[3] Ferris RL , Blumenschein G Jr, Fayette J, Guigay J, Colevas AD, Licitra L, . Nivolumab for recurrent squamous-cell carcinoma of the head and neck. N Engl J Med 2016;375:1856–67. 27718784 10.1056/NEJMoa1602252 PMC5564292

[4] Motzer RJ , Escudier B, McDermott DF, George S, Hammers HJ, Srinivas S, . Nivolumab versus everolimus in advanced renal-cell carcinoma. N Engl J Med 2015;373:1803–13. 26406148 10.1056/NEJMoa1510665 PMC5719487

[5] Kang Y-K , Boku N, Satoh T, Ryu M-H, Chao Y, Kato K, . Nivolumab in patients with advanced gastric or gastro-oesophageal junction cancer refractory to, or intolerant of, at least two previous chemotherapy regimens (ONO-4538-12, ATTRACTION-2): a randomised, double-blind, placebo-controlled, phase 3 trial. Lancet 2017;390:2461–71. 28993052 10.1016/S0140-6736(17)31827-5

[6] Robert C , Long GV, Brady B, Dutriaux C, Maio M, Mortier L, . Nivolumab in previously untreated melanoma without BRAF mutation. N Engl J Med 2015;372:320–30. 25399552 10.1056/NEJMoa1412082

[7] Bristol Myers Squibb . OPDIVO® (nivolumab) [package insert; Internet]. Princeton (NJ): Bristol Myers Squibb. [cited 2024 Sep 19]. Available from: https://packageinserts.bms.com/pi/pi_opdivo.pdf.

[8] Bristol Myers Squibb . OPDIVO® (nivolumab) [summary of product characteristics; Internet]. Dublin (Ireland): Bristol Myers Squibb. [cited 2024 Sep 19]. Available from: https://www.ema.europa.eu/en/documents/product-information/opdivo-epar-product-information_en.pdf.

[9] Postow MA , Sidlow R, Hellmann MD. Immune-related adverse events associated with immune checkpoint blockade. N Engl J Med 2018;378:158–68. 29320654 10.1056/NEJMra1703481

[10] Marabelle A , Le DT, Ascierto PA, Di Giacomo AM, De Jesus-Acosta A, Delord J-P, . Efficacy of pembrolizumab in patients with noncolorectal high microsatellite instability/mismatch repair-deficient cancer: results from the phase II KEYNOTE-158 study. J Clin Oncol 2020;38:1–10. 31682550 10.1200/JCO.19.02105 PMC8184060

[11] Le DT , Kim TW, Van Cutsem E, Geva R, Jäger D, Hara H, . Phase II open-label study of pembrolizumab in treatment-refractory, microsatellite instability-high/mismatch repair-deficient metastatic colorectal cancer: KEYNOTE-164. J Clin Oncol 2020;38:11–9. 31725351 10.1200/JCO.19.02107 PMC7031958

[12] Geoerger B , Kang HJ, Yalon-Oren M, Marshall LV, Vezina C, Pappo AS, . KEYNOTE-051: an update on the phase 2 results of pembrolizumab (pembro) in pediatric patients (pts) with advanced melanoma or a PD-L1–positive advanced, relapsed or refractory solid tumor or lymphoma. J Clin Oncol 2018;36(Suppl 15):10525. 10.1016/S1470-2045(19)30671-0 31812554

[13] Riely GJ , Wood DE, Ettinger DS, Aisner DL, Akerley W, Bauman JR, . Non-small cell lung cancer, version 4.2024, NCCN clinical practice guidelines in oncology. J Natl Compr Canc Netw 2024;22:249–74. 38754467 10.6004/jnccn.2204.0023

[14] The Japan Lung Cancer Society . Guidelines for the diagnosis and treatment of lung cancer [Internet]. The Japan Lung Cancer Society. [cited 2024 Sep 4]. Available from: https://www.haigan.gr.jp/publication/guideline/examination/2023/.

[15] Catalano M , Iannone LF, Nesi G, Nobili S, Mini E, Roviello G. Immunotherapy-related biomarkers: confirmations and uncertainties. Crit Rev Oncol Hematol 2023;192:104135. 37717881 10.1016/j.critrevonc.2023.104135

[16] Sharma P , Callahan MK, Bono P, Kim J, Spiliopoulou P, Calvo E, . Nivolumab monotherapy in recurrent metastatic urothelial carcinoma (CheckMate 032): a multicentre, open-label, two-stage, multi-arm, phase 1/2 trial. Lancet Oncol 2016;17:1590–8. 27733243 10.1016/S1470-2045(16)30496-X PMC5648054

[17] Garon EB , Rizvi NA, Hui R, Leighl N, Balmanoukian AS, Eder JP, . Pembrolizumab for the treatment of non–small-cell lung cancer. N Engl J Med 2015;372:2018–28. 25891174 10.1056/NEJMoa1501824

[18] European Medicines Agency . Assessment report. Keytruda. Amsterdam (the Netherlands): European Medicines Agency; 2016.

[19] Janjigian YY , Shitara K, Moehler M, Garrido M, Salman P, Shen L, . First-line nivolumab plus chemotherapy versus chemotherapy alone for advanced gastric, gastro-oesophageal junction, and oesophageal adenocarcinoma (CheckMate 649): a randomised, open-label, phase 3 trial. Lancet 2021;398:27–40. 34102137 10.1016/S0140-6736(21)00797-2 PMC8436782

[20] Kulangara K , Zhang N, Corigliano E, Guerrero L, Waldroup S, Jaiswal D, . Clinical utility of the combined positive score for programmed death ligand-1 expression and the approval of pembrolizumab for treatment of gastric cancer. Arch Pathol Lab Med 2019;143:330–7. 30028179 10.5858/arpa.2018-0043-OA

[21] Thommen DS , Koelzer VH, Herzig P, Roller A, Trefny M, Dimeloe S, . A transcriptionally and functionally distinct PD-1^+^ CD8^+^ T cell pool with predictive potential in non-small-cell lung cancer treated with PD-1 blockade. Nat Med 2018;24:994–1004. 29892065 10.1038/s41591-018-0057-z PMC6110381

[22] Kansy BA , Concha-Benavente F, Srivastava RM, Jie H-B, Shayan G, Lei Y, . PD-1 status in CD8^+^ T cells associates with survival and anti-PD-1 therapeutic outcomes in head and neck cancer. Cancer Res 2017;77:6353–64. 28904066 10.1158/0008-5472.CAN-16-3167 PMC5690836

[23] Gros A , Robbins PF, Yao X, Li YF, Turcotte S, Tran E, . PD-1 identifies the patient-specific CD8^+^ tumor-reactive repertoire infiltrating human tumors. J Clin Invest 2014;124:2246–59. 24667641 10.1172/JCI73639 PMC4001555

[24] Togashi Y , Shitara K, Nishikawa H. Regulatory T cells in cancer immunosuppression — implications for anticancer therapy. Nat Rev Clin Oncol 2019;16:356–71. 30705439 10.1038/s41571-019-0175-7

[25] Kamada T , Togashi Y, Tay C, Ha D, Sasaki A, Nakamura Y, . PD-1^+^ regulatory T cells amplified by PD-1 blockade promote hyperprogression of cancer. Proc Natl Acad Sci U S A 2019;116:9999–10008. 31028147 10.1073/pnas.1822001116 PMC6525547

[26] Kumagai S , Togashi Y, Kamada T, Sugiyama E, Nishinakamura H, Takeuchi Y, . The PD-1 expression balance between effector and regulatory T cells predicts the clinical efficacy of PD-1 blockade therapies. Nat Immunol 2020;21:1346–58. 32868929 10.1038/s41590-020-0769-3

[27] Eisenhauer EA , Therasse P, Bogaerts J, Schwartz LH, Sargent D, Ford R, . New response evaluation criteria in solid tumours: revised RECIST guideline (version 1.1). Eur J Cancer 2009;45:228–47. 19097774 10.1016/j.ejca.2008.10.026

[28] Spagnolo F , Boutros A, Cecchi F, Croce E, Tanda ET, Queirolo P. Treatment beyond progression with anti-PD-1/PD-L1 based regimens in advanced solid tumors: a systematic review. BMC Cancer 2021;21:425. 33865350 10.1186/s12885-021-08165-0 PMC8052683

[29] Kobayashi T , Kumagai S, Doi R, Afonina E, Koyama S, Nishikawa H. Isolation of tumor-infiltrating lymphocytes from preserved human tumor tissue specimens for downstream characterization. STAR Protoc 2022;3:101557. 35852944 10.1016/j.xpro.2022.101557 PMC9304678

[30] FoundationOne®CDx . Technical information [Internet]. Cambridge (MA): Foundation Medicine, Inc. [cited 2024 Oct 2]. Available from: https://www.accessdata.fda.gov/scripts/cdrh/cfdocs/cfpma/pma.cfm?id=P170019S029.pdf.

[31] DAKO Agilent Technologies . PD-L1 IHC 28-8 pharmDx [Internet]. Santa Clara (CA): DAKO Agilent Technologies. [cited 2024 Oct 2]. Available from: https://www.accessdata.fda.gov/cdrh_docs/pdf15/P150025S013C.pdf.

[32] Borghaei H , Gettinger S, Vokes EE, Chow LQM, Burgio MA, de Castro Carpeno J, . Five-year outcomes from the randomized, phase III trials checkmate 017 and 057: nivolumab versus docetaxel in previously treated non-small-cell lung cancer. J Clin Oncol 2021;39:723–33. 33449799 10.1200/JCO.20.01605 PMC8078445

[33] Herbst RS , Garon EB, Kim D-W, Cho BC, Gervais R, Perez-Gracia JL, . Five year survival update from KEYNOTE-010: pembrolizumab versus docetaxel for previously treated, programmed death-ligand 1–positive advanced NSCLC. J Thorac Oncol 2021;16:1718–32. 34048946 10.1016/j.jtho.2021.05.001

[34] De Castro G Jr , Kudaba I, Wu Y-L, Lopes G, Kowalski DM, Turna HZ, . Five-year outcomes with pembrolizumab versus chemotherapy as first-line therapy in patients with non-small-cell lung cancer and programmed death ligand-1 tumor proportion score ≥1% in the KEYNOTE-042 study. J Clin Oncol 2023;41:1986–91. 36306479 10.1200/JCO.21.02885 PMC10082298

[35] Shitara K , Özgüroğlu M, Bang Y-J, Di Bartolomeo M, Mandalà M, Ryu M-H, . Pembrolizumab versus paclitaxel for previously treated, advanced gastric or gastro-oesophageal junction cancer (KEYNOTE-061): a randomised, open-label, controlled, phase 3 trial. Lancet 2018;392:123–33. 29880231 10.1016/S0140-6736(18)31257-1

[36] Shitara K , Van Cutsem E, Bang Y-J, Fuchs C, Wyrwicz L, Lee K-W, . Efficacy and safety of pembrolizumab or pembrolizumab plus chemotherapy vs chemotherapy alone for patients with first-line, advanced gastric cancer: the KEYNOTE-062 phase 3 randomized clinical trial. JAMA Oncol 2020;6:1571–80. 32880601 10.1001/jamaoncol.2020.3370 PMC7489405

[37] Carbone DP , Reck M, Paz-Ares L, Creelan B, Horn L, Steins M, . First-line nivolumab in stage IV or recurrent non–small-cell lung cancer. N Engl J Med 2017;376:2415–26. 28636851 10.1056/NEJMoa1613493 PMC6487310

[38] Narita Y , Matsushima T, Sakamoto Y, Matsuoka H, Tanioka H, Kawakami T, . Chemotherapy after nivolumab for advanced gastric cancer (REVIVE): a prospective observational study. ESMO Open 2023;8:102071. 38016249 10.1016/j.esmoop.2023.102071 PMC10774960

[39] Reck M , Rodríguez-Abreu D, Robinson AG, Hui R, Csőszi T, Fülöp A, . Pembrolizumab versus chemotherapy for PD-L1–positive non–small-cell lung cancer. N Engl J Med 2016;375:1823–33. 27718847 10.1056/NEJMoa1606774

[40] Mok TSK , Lopes G, Cho BC, Kowalski DM, Kasahara K, Wu Y-L, . Associations of tissue tumor mutational burden and mutational status with clinical outcomes in KEYNOTE-042: pembrolizumab versus chemotherapy for advanced PD-L1-positive NSCLC. Ann Oncol 2023;34:377–88. 36709038 10.1016/j.annonc.2023.01.011

[41] Fuchs CS , Doi T, Jang RW, Muro K, Satoh T, Machado M, . Safety and efficacy of pembrolizumab monotherapy in patients with previously treated advanced gastric and gastroesophageal junction cancer: phase 2 clinical KEYNOTE-059 trial. JAMA Oncol 2018;4:e180013. 29543932 10.1001/jamaoncol.2018.0013 PMC5885175

[42] Overman MJ , McDermott R, Leach JL, Lonardi S, Lenz H-J, Morse MA, . Nivolumab in patients with metastatic DNA mismatch repair-deficient or microsatellite instability-high colorectal cancer (CheckMate 142): an open-label, multicentre, phase 2 study. Lancet Oncol 2017;18:1182–91. 28734759 10.1016/S1470-2045(17)30422-9 PMC6207072

[43] Yoshida T , Ogura G, Tanabe M, Hayashi T, Ohbayashi C, Azuma M, . Clinicopathological features of PD-L1 protein expression, EBV positivity, and MSI status in patients with advanced gastric and esophagogastric junction adenocarcinoma in Japan. Cancer Biol Ther 2022;23:191–200. 35220884 10.1080/15384047.2022.2038002 PMC8890430

[44] Ribeiro MB , Marques SB, Soares IC, Pereira MA, Takeda FR, Safatle-Ribeiro AV, . Epstein-Barr virus and PD-L1 in esophageal and esophagogastric junction cancer: differences according to location and histological type. J Gastrointest Surg 2022;26:2358–64. 35668227 10.1007/s11605-022-05377-y

