# Supplementary material for: Efficacy and Safety of Nivolumab Monotherapy in Patients with High PD-1–Positive CD8/Treg Ratio in Advanced NSCLC and Gastric Cancer: A Phase II, Multicenter Study
Source: Cancer Res Commun. 2025 Oct 13;5(10):1809–20. doi: 10.1158/2767-9764.CRC-25-0169 (PMC12525050; doi:10.1158/2767-9764.CRC-25-0169)
Supplement: Supplementary Table S1 — The antibodies list used for TIL analysis [file crc-25-0169_supplementary_table_s1_suppst1.docx]

**Supplementary Table S1. The antibodies list used for TIL analysis**

| **Tube** | **Antibody** | **Fluorochrome** | **Clone** | **Isotype** |
| --- | --- | --- | --- | --- |
| Tube 1 | CD3 | V500-C | SK7 | IgG1, κ |
|  | CD4 | PerCP-Cy5.5 | SK3 | IgG1, κ |
|  | CD45RA | PE-Cy7 | L48 | IgG1, κ |
|  | CD8 | FITC | SK1 | IgG1, κ |
|  | PD1 | BV421 | MIH4 | IgG1, κ |
| Tube 2 | FoxP3 | PE | 236A/E7 | IgG1, κ |
| Isotype Control | CD3 | V500-C | SK7 | IgG1, κ |
|  | CD4 | PerCP-Cy5.5 | SK3 | IgG1, κ |
|  | CD45RA | PE-Cy7 | L48 | IgG1, κ |
|  | CD8 | FITC | SK1 | IgG1, κ |
|  | Mouse IgG1 | BV421 | X40 | IgG1, κ |
