# Supplementary material for: Efficacy and Safety of Nivolumab Monotherapy in Patients with High PD-1–Positive CD8/Treg Ratio in Advanced NSCLC and Gastric Cancer: A Phase II, Multicenter Study
Source: Cancer Res Commun. 2025 Oct 13;5(10):1809–20. doi: 10.1158/2767-9764.CRC-25-0169 (PMC12525050; doi:10.1158/2767-9764.CRC-25-0169)
Supplement: Supplementary Table S2 — Representativeness of study participants [file crc-25-0169_supplementary_table_s2_suppst2.docx]

**Supplementary Table S2. Representativeness of study participants**

| **Category** | **This study** |
| --- | --- |
| **Cancer type and condition** | Recurrent or stage IIIB/IIIC/IV non-small cell lung cancer (NSCLC) unsuitable for definitive radiation, or those with unresectable advanced or recurrent gastric cancer (GC) |
| **Considerations related to:** | |
| Sex | The majority of patients in this study were male (5/5 patients [100.0%] with NSCLC and 7/13 patients [53.8%] with GC). This trend was similar to findings in the 2022 GLOBOCAN report, which included 95,740 male and 40,983 female with NSCLC and 84,071 male and 42,653 female with GC in Japan.^a^ |
| Age | The median age of patients in this study was 75.0 years for NSCLC and 74.0 years for GC. These are consistent with the real-world patient population in Japan (median age; 70 for NSCLC and 67 for GC).^b,c^ |
| Race/ethnicity/geography | All patients in this study were Japanese. In Japan, NSCLC and GC are among the top 5 cancers with high incidence, mortality, and prevalence.^a^ |
| **Overall representativeness of this study** | Despite the numerically high variability due to limited number of patients in this study, overall characteristics of patients in the present study are generally consistent with Japanese adult patients who were diagnosed with NSCLC and GC. For our findings, further validation would be required by enrolling a larger size of patients from different countries. |

^a^ Japan – Global Cancer Observatory. GLOBOCAN 2022. Accessed February 19, 2025.

<https://gco.iarc.who.int/media/globocan/factsheets/populations/392-japan-fact-sheet.pdf>

^b^ Nokihara H, Kijima T, Yokoyama T, et al. Real-World Treatments and Clinical Outcomes in Advanced NSCLC without Actionable Mutations after Introduction of Immunotherapy in Japan. *Cancers*. **2022**;14(12):2846.

^c^ Katai H, Ishikawa T, Akazawa K, et al. Five-year survival analysis of surgically resected gastric cancer cases in Japan: a retrospective analysis of more than 100,000 patients from the nationwide registry of the Japanese Gastric Cancer Association (2001-2007). *Gastric Cancer*. **2018**;21:144–154.
