# Supplementary material for: Efficacy and Safety of Nivolumab Monotherapy in Patients with High PD-1–Positive CD8/Treg Ratio in Advanced NSCLC and Gastric Cancer: A Phase II, Multicenter Study
Source: Cancer Res Commun. 2025 Oct 13;5(10):1809–20. doi: 10.1158/2767-9764.CRC-25-0169 (PMC12525050; doi:10.1158/2767-9764.CRC-25-0169)
Supplement: Supplementary Table S3 — Post-progression anti-cancer therapy of the nivolumab monotherapy [file crc-25-0169_supplementary_table_s3_suppst3.docx]

**Supplementary Table S3.** **Post-progression anti-cancer therapy of the nivolumab monotherapy**

| Analysis set: FAS | NSCLC |  | Analysis set: FAS | GC |
| --- | --- | --- | --- | --- |
|  | N = 5 |  |  | N = 13 |
|  | n* (%) |  |  | n* (%) |
| Any subsequent anti-cancer therapy | 2 (40.0) |  | Any subsequent anti-cancer therapy | 6 (46.2) |
| Cytotoxic Chemotherapy Alone | 1** (20.0) |  | Cytotoxic Chemotherapy Alone | 4*** (30.8) |
| Pemetrexed + Carboplatin | 1 (20.0) |  | SOX | 4 (30.8) |
| Tegafur + Gimerasil + Oterasil | 1 (20.0) |  | FOLFOX | 1 (7.7) |
| Targeted Therapy + Cytotoxic Chemotherapy | 2** (40.0) |  | Paclitaxel | 1 (7.7) |
| Bevacizumab + nab-Paclitaxel | 1 (20.0) |  | Trifluridine + Tipiracil | 1 (7.7) |
| Bevacizumab + Pemetrexed + Carboplatin | 1 (20.0) |  | Irinotecan + Cisplatin | 1 (7.7) |
| Ramucirumab + Docetaxel | 1 (20.0) |  | Targeted Therapy + Cytotoxic Chemotherapy | 3*** (23.1) |
| No subsequent anti-cancer therapy | 3 (60.0) |  | Trastuzumab + SOX | 1 (7.7) |
|  |  |  | Ramucirumab + Paclitaxel | 1 (7.7) |
|  |  |  | Ramucirumab + nab-Paclitaxel | 1 (7.7) |
|  |  |  | Immunotherapy + Cytotoxic Chemotherapy | 1 (7.7) |
|  |  |  | Nivolumab + SOX | 1 (7.7) |
|  |  |  | No subsequent anti-cancer therapy | 7 (53.8) |

*Includes duplication.

**One patient received both combination of cytotoxic chemotherapy and targeted therapy plus cytotoxic chemotherapy.

***Two patients received both combination of cytotoxic chemotherapy and targeted therapy plus cytotoxic chemotherapy.

FAS, full analysis set; FOLFOX, combination therapy of fluorouracil, leucovorin, and oxaliplatin; SOX, combination therapy of S-1 plus oxaliplatin.
